# Microbial Cells as a Microrobots: From Drug Delivery to Advanced Biosensors

**DOI:** 10.3390/biomimetics8010109

**Published:** 2023-03-07

**Authors:** Pavel Gotovtsev

**Affiliations:** 1 National Research Center “Kurchatov Institute”, Biotechnology and Bioenergy Department, Akademika Kurchatova pl. 1, 123182 Moscow, Russia; gotovtsevpm@gmail.com; 2 Moscow Institute of Physics and Technology, National Research University, 9 Institutskiy per., 141701 Moscow, Russia

**Keywords:** biohybrid microrobots, microorganism motility, immobilization, synthetic biology, nanoparticles, xenobiology

## Abstract

The presented review focused on the microbial cell based system. This approach is based on the application of microorganisms as the main part of a robot that is responsible for the motility, cargo shipping, and in some cases, the production of useful chemicals. Living cells in such microrobots have both advantages and disadvantages. Regarding the advantages, it is necessary to mention the motility of cells, which can be natural chemotaxis or phototaxis, depending on the organism. There are approaches to make cells magnetotactic by adding nanoparticles to their surface. Today, the results of the development of such microrobots have been widely discussed. It has been shown that there is a possibility of combining different types of taxis to enhance the control level of the microrobots based on the microorganisms’ cells and the efficiency of the solving task. Another advantage is the possibility of applying the whole potential of synthetic biology to make the behavior of the cells more controllable and complex. Biosynthesis of the cargo, advanced sensing, on/off switches, and other promising approaches are discussed within the context of the application for the microrobots. Thus, a synthetic biology application offers significant perspectives on microbial cell based microrobot development. Disadvantages that follow from the nature of microbial cells such as the number of external factors influence the cells, potential immune reaction, etc. They provide several limitations in the application, but do not decrease the bright perspectives of microrobots based on the cells of the microorganisms.

## 1. Introduction

**Figure 1:**
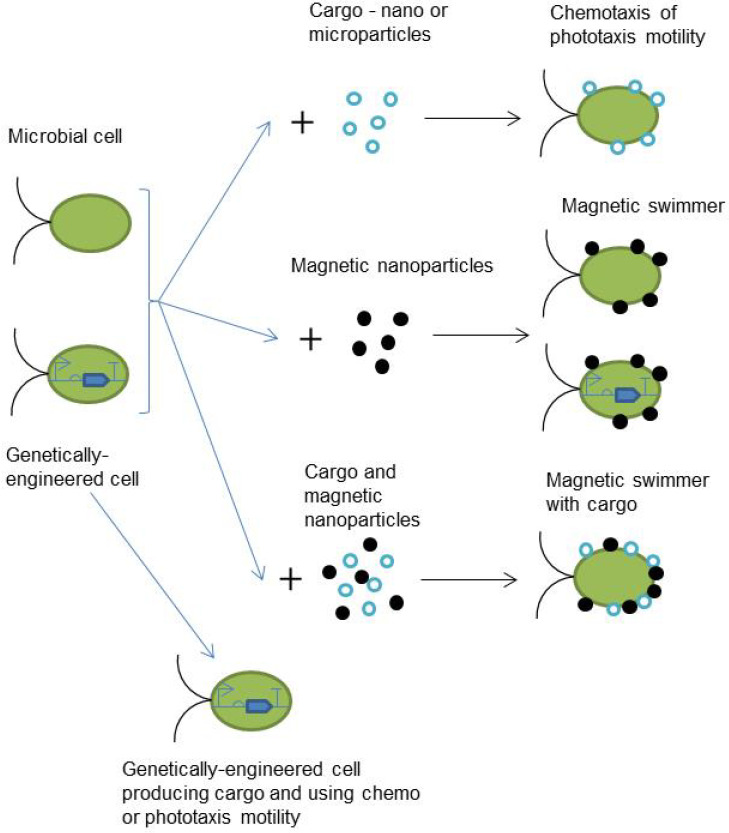
Most of the discussed variants of the microrobot design based on microbial cells.

Today, the development of microrobots is a highly dynamic field of research [1,2,3]. There are numerous different approaches presented in scientific literature [4,5]: magnetic swimmers [6,7] and inchworms [8], optical driven microrobots [9,10], lyposome-based systems [11], enzyme catalysis powered micromotors [12], and many others. Among them, microorganisms are also a focus of interest because microbial cells are already mobile systems with high levels of autonomy as they have already an “energy system”, can use chemicals from the environment as food, use chemical or sunlight as an energy source, replicate itself via division, provide the biosynthesis of necessary chemicals, and demonstrate directional movement. Today, genetic engineering and synthetic biology approaches offer many different possibilities to program cell behavior and even to organize simple logic circuits inside the cell for data processing based on the design of genetic logic circuits [13,14]. Different nano- and microparticles can be added to the cells as a cargo that should be transferred somewhere or as a part of the actuating or control system [15,16,17]. Figure 1 shows the most discussed variants of the design of microrobots based on microbial cells. Thus, on one hand, there is a perspective to take the already existing natural system and adapt it to the solving of practical tasks at the micro level. On the other hand, the complexity of the living system makes the development of such microrobots very challenging. The operation of microbial robots is the dynamical system of billions of cells, and mostly, the number of cells is not constant [18]. This dynamic system exists in a very changeable environment and interacts with it in many ways. In the future, such microrobots will be used in different fields but limited by the environment. For example, compatibility and the absence of any interactions except those desired are necessary in the case of medical applications inside the human body [1].

The goal of this review was to analyze the published results on microbial cell microrobot development and discuss perspectives of this technology with respect to the current advances in synthetic biology and related fields.

## 2. Current Approaches in the Microbial Cell Based Microrobots Development

One of the advantages of microbial cells is the already existing motility system. Depending on the strain, chemotaxis [19], phototaxis [20], and magnetotaxis [21] can be used. Additionally, electrotaxis can possibly be discussed in the future. Today, this type of motility is studied for mammalian cell lines [22], but very little is known about the electrotaxis of microorganisms [23].

### 2.1. Chemotaxis-Based Microrobots

Chemotaxis is based on the sensing of attractive chemicals (attractants) in the medium, which can be useful for the life cycle of further microorganisms [24]. Typical objects of chemotaxis studies are different bacteria species [24]. It is necessary to mention that in nature, chemotaxis mostly leads to the formation of a biofilm in the end [25]. There are several significant features related to chemotaxis that are necessary to take into account from the robotics application point of view:

1. The chemotaxis process is highly expensive for bacteria, for example, it requires around 3% of the total protein amount in *Escherichia Coli* [26];
2. Bacterial cells do not move as a number of standalone agents using chemotaxis—they have a mechanism of cell-to-cell chemical communication by secreting and sensing small molecules in the environment, which is named quorum sensing [27];
3. Both chemotaxis and quorum sensing take place in the liquid media, thus, diffusion is low and hydrodynamics can make such signals noisy [19]; and
4. Even in the clonal population of the bacteria, chemotactic sensitivity can be different [28].

This feature leads to some effects during the motion and distribution of bacteria cells in the media. Not all bacteria cells will be at the point of interest (the maximum concentration of the attractant). It is necessary to take into account this phenomenon in the case of the development of microrobots to solve the drug delivery task. For example, if the carried drug focused on the cancer cells is toxic for normal cells, such a distribution of bacteria should be strongly analyzed. Chemotaxis is described mathematically using several approaches [29,30,31]. The most common model is the Keller–Segel framework [32,33,34], which can be described in general by the next equations [29]:

(1) $$\frac{\partial b}{\partial t}=\nabla \left(\mu \left(s\right)\nabla b\right)-\nabla \left(\chi \left(s\right)b\nabla s\right)+g\left(b,s\right)-h\left(b,s\right)$$

(2) $$\frac{\partial s}{\partial t}=D\nabla^{2}s-f\left(b,s\right)$$

where b—bacterial population density, which can be described as b = b(x,t); s—concentration of the attractant, which also can be described as s = s(x,t); x—dimensional position; t—time; $\mu \left(s\right)$—coefficient that describes bacterial diffusion; $\chi \left(s\right)$—coefficient that describes chemotaxis; $g\left(b,s\right)$—cell growth function; $h\left(b,s\right)$—cell death function; $f\left(b,s\right)$—presents attractant degradation; D—diffusion coefficient. The experiment environment and physical model define the boundary conditions. There are several methods to simplify Equations (1) and (2). First, it can be assumed that bacterial diffusion does not change in the space of the experiment, thus, $\mu \left(s\right)=\mu$. Second, assume that the speed of cell death and cell growth is constant in a given medium. For example, discuss only one phase of cell growth such as the exponential part. These two assumptions are very weak and can be applied in laboratory experiments in controlled environments. However, they should be strongly discussed in the case of application for close-to-real tests. Nevertheless, chemotaxis-like models have discussed as one of the most promising mechanisms for the behavior control of a group of robots in the adapted form [35,36].

Kim et al. reported [18] that *Serratia marcescens* bacteria were used as a carrier of polystyrene microbeads. It was shown that it is possible to move a significant number of cells with a microbead cargo to the source of the attractant in the microfluidic device using chemotaxis mechanisms.

The addition of the cargo at the bacterial cell can influence its motility ability. Shauer et al. reported [15] that the swimming speed of *E. coli* was reduced from 15.72 ± 0.02 μm/s to 9.76 ± 0.02 μm/s upon the attachment of 2.2 μm particles. The authors showed that the cephalexin treatment led to the increase in the cell length and decrease in its speed without load. However, the changes in speed were not as significant as those for the untreated cells in the case of the addition of particles. The motility was also the result of chemotaxis of the bacterial cells.

Oxygen can be a strong attractant for some microorganisms. Shechter et al. [37] demonstrated that bacteria actively moved via oxygen gradient in the medium from the lowest concentration to the highest. It is necessary to mention that magnetotactic bacteria were used in this study, which provides an opportunity to investigate joint control by using magnetic field and oxygen concentration gradients.

Several bacteria cells can move a bigger object, as demonstrated by Higashi et al. [38]. In this study, a microarray with holes 200 μm deep and 130 μm in diameter at the inlet side was developed. Then, a higher concentration of oxygen was established in those holes. The bacteria *Gluconacetobacter xylinus* demonstrated chemotaxis motility in the direction of higher oxygen concentration in the medium. Thus, they moved in the holes of the microarray. *G. xylinus* was chosen because this bacterium is a producer of bacterial cellulose [39]. Thus, bacterial cellulose structures were synthesized in the holes of the microarray. Next, flagellated bacteria *Aliivibrio fischeri* cells were immobilized on the only available side of the bacteria cellulose structure [38]. The resulting microrobot had the longest dimension of more than 200 μm and could be moved in the medium by the joint forces of several bacteria cells. The motility speed of the microrobot was around 4.8 μm/s, which is significantly slower than the motility speed of *A. fischeri* cells (around 50 μm/s). In spite of such a difference in speed, the possibility of creating hundreds of micrometer long moving structures from the biopolymers provides large opportunities to build more complex biorobotic systems operating at the microscale level.

### 2.2. Phototaxis-Based Microrobots

Phototaxis motility demonstrated by phototrophic microorganisms has been less investigated in comparison with chemotaxis. Depending on the intensity of illumination and intracellular processes, phototaxis can be directed to the light source and away from it; in this way, microorganisms can find more favorable conditions [20,40]. Motility speed depends on the cell concentration as shown for *Chlamydomonas reinhardtii* [41]. For high concentrations around 10^8^ cells/cm^−3^, it can be as low as 100 μm/s, then for concentrations around 10^6^ cells/cm^−3^, the motility speed can be up to 130 μm/s. *C. reinhardtii* is a microalga with two flagella. It is commonly used as a model organism for a number of studies related to phototrophic microorganisms including phototaxis [40,42,43]. Both flagella are placed on one side of the cell, thus the rotation frequency also influences the motility overall. It was shown that cell concentration also influences rotation frequency in the same manner as the motility speed [41]. The maximum magnitude was close to 1.8 Hz for a concentration about 10^6^ cells/cm^−3^, and less for higher concentrations—1.2 Hz for 10^8^ cells/cm^−3^. Several simulations of phototaxis showed that the adapted Keller–Segel framework showed a good correlation with the laboratory modeling experiments [20,44,45].

*C. reinhardtii* was used as the biological part of a biohybrid microrobot that carried nanoparticles as a cargo [16]. Chitosan’s soft coating allows for the use of different cargoes without a negative impact on the viability and phototactic ability of microalgae. The average speed of biohybrid cells was less in comparison with native culture, 56.3 ± 1.1 μm/s and 109.2 ± 1 μm/s, respectively. Cargo delivery was demonstrated by using the model cancer drug doxorubicin (DOX) with photocleavable sites. Experiments with breast cancer cell culture SK-BR-3 demonstrated effective drug delivery and release by biohybrid cells.

Precise control of phototaxis motility requires a feedback loop. Optical or fluorescent microscopy is usually used as a source of feedback signal [20]. Xie et al. [46] presented a microscope-based system for the controlled locomotion of algae cells. Input signal was a varying light from light emitted diodes generated based on the feedback signal, and goals were given by the operator. Algae *Eudorina elegans* was used as the subject of the experiment. Despite this, *E. elegans* is a multicellular algae that is very small, with sizes from 10 to 200 μm depending on the living cycle. The effective control of such algae motility presented by the technique in this paper offers intriguing perspectives. It would be interesting to provide such experiments with *Arthrospira platensis*—a filamentous cyanobacterium. The length of filaments formed by this phototrophic microorganism is hundreds of μm depending on the culture conditions [47]. *A. platensis* is the natural producer of C-phycocyanin and is a promising anticancer drug [48]. This cyanobacterium is already used in the food industry [47].

The fast speed of *C. reinhardtii* motility makes this strain prospective for application without phototaxis. In the appropriate environmental conditions, the cells were distributed in almost all of the given volumes. Zhand et al. [49] demonstrated such an effect during the injection in the lungs of laboratory mice. The cell surface was modified to carry nanoparticles with a cargo by using methods of click chemistry. In the experiments with animals, antibiotic ciprofloxacin was used to prevent bacterial infection. The addition of microrobots to the infected lungs led to a significant decrease in the bacteria population. It is necessary to mention that the method of adding cargo at the cell wall presented in the paper did not influence the motility speed and stayed the same for algae and the microrobot and equal to 115.5 ± 11.8 μm/s.

*C. reinhardtii* was used both as a carrier of therapeutic drugs and as an oxygen producer to treat diabetic wounds [50]. Interleukin-8 and monocyte chemoattractant protein-1 for immune modulation were integrated into the chitosan–heparin nanocomplex that was immobilized in the cell wall. Experiments provided on the real wound on the laboratory mice demonstrated that part of the microbot penetrated through the blood clots and part stayed in the wound site. The addition of microrobots led to the wound healing in 9 days and took more than 12 days for the control wounds.

### 2.3. Magnetotaxis-Based Microrobots

Interest in magnetotactic microorganisms as a basis for microrobots is based on the possibility of using magnetic resonance imaging (MRI) to track and control it inside the human body, especially in the vascular system [51]. There are two approaches in the design of magnetically driven biohybrid microrobots that can be discussed: using naturally magnetotactic bacteria [21], or adding magnetic nanoparticles to bacteria or microalga, making them magnetotactic [52]. Natural magnetotactic bacteria are able to provide a synthesis of magnetic nanoparticles inside the cell in the magnetosomes [21]. These bacteria can navigate in the Earth’s magnetic field, moving along field lines [53]. Magnetotactic bacteria are usually difficult to handle on a significant scale and the main practical applications focus on the biosynthesis of magnetic nanoparticles [21]. Nevertheless, the possibility of transferring 3 μm cells with a speed equal to 7.5 μm/s has been demonstrated [54]. Although magnetotactic bacteria themselves have not been discussed yet as a biological basis for microrobots, the mechanisms of the biosynthesis of intracellular magnetic nanoparticles is of considerable interest. The addition oof magnetic nanoparticles on the cell-wall of the microalgae do not offer the possibility to translate the magnetotactic ability to the next generations. Thus, the expression of magnetosome-related genes in more easy-to-handle microorganisms can will be a significant future challenge.

The addition of magnetic microparticles (around 1 μm) influenced the mean motility speed of *C. reinhardtii* microalgae from 109.54 ± 2.59 μm/s to 51.89 ± 1.89 μm/s in 2D conditions [52]. In the case of the application of a uniform magnetic field, the mean motility speed was equal to 51.44 ± 2.16 μm/s. It was mentioned that cells lose the ability to move if microparticles are placed on the flagella section.

DOX with magnetic nanoparticles were incorporated in the red blood cells, which in its turn was linked with *E. coli* via the biotin–avidin–biotin complex [55]. Although the red blood cell was bigger than *E. coli*, the whole microrobot demonstrated motility with a speed equal to 10.2 ± 3.5 μm/s. The role of the bacteria cell was to enhance the swimming capability of the whole system, especially in complex microchannels where flagellated bacteria can ensure autonomous movements to prevent jamming. The release of DOX was faster in lower pH—more than 70% was released in 3 h at pH = 3.1 and more than 40% at pH = 7.2. A microrobot with a size of more than 4 μm demonstrated the ability to move through the microchannels with a width of 3 μm because of its own deformation. It is important to note that indocyanine green was added into the red blood cells. Upon irradiation in near infrared light, this molecule leads to hyperthermia, rupture of the blood cell membrane, and the death of bacteria.

*Volvox aureus* green multicellular alga was used to create biohybrid microrobots that were capable of transporting anticancer drugs as cargo and produced oxygen for hypoxia prevention [56]. The cargo system was based on the functionalization of the target drug or nanoparticles by chitosan. The advantage of this microrobot is in the combination of two motility approaches: phototaxis as a natural ability of alga, and magnetotaxis via the addition of magnetic nanoparticles in the cargo. Red light (650 nm) was used to the control the phototactic rotation and locomotion. Due to photosynthesis, the concentration of dissolved oxygen in the target area could reach 18.78 ppm. The maximum microrobot speed was close to 400 μm/s.

### 2.4. Comparison of the Different Motility Types

**Figure 2:**
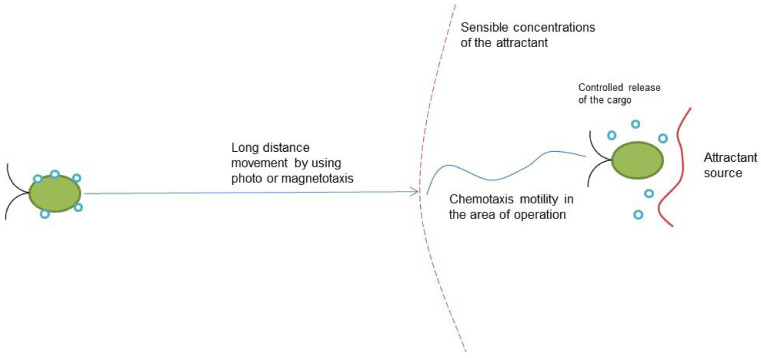
Variant of the application of the two motility types for the microrobot.

Table 1 summarizes the information on the motility of the microrobots. Electrotaxis was not included because of the absence of microrobot projects using it and the lack of scientific data concerning bacterial electrotaxis. Each type of motility has its own advantages and limitations that influence the prospective application of microrobots. Chemotaxis is the only type of motility that can be realized in a closed environment without external stimulus. The system biology of chemotaxis is quite well-studied [57], as has been the structure of flagella for some strains [58]. Phototaxis is inherent to the phototrophic microorganisms that can produce oxygen via photosynthesis—the significant ability to prevent hypoxia that is related to many illnesses. Additionally, some microalgae have a very large motility speed without cargo. The combination of different types of motility could be a very promising approach for the development of future microrobots. For example, magnetotaxis can be used to move microrobots into the area of operation via MRI and chemotaxis for motility in this area (Figure 2). Such combinations can significantly expand their possible fields of application.

Magnetotaxis can be easily accomplished for non-magnetotactic microorganisms by adding magnetic nanoparticles, but this ability is not inherited through the generations of cells. However, in most cases, the therapeutic properties of microrobots are related to the cargo and also are not inherited through the generations. Thus, most of the discussed microrobots are single-use and the ability of the microorganisms to replicate themselves has not been considered in most of the discussed projects. Next, synthetic biology approaches will be discussed as a prospective way to enhance microbial cell-based microrobots with the focus on the replication of with the necessary capabilities.

## 3. Synthetic Biology Approaches for Microbial Cell-Based Microrobots Design

The application of genetically engineered microorganisms in medicine, industrial monitoring, and environmental monitoring are being actively discussed today [59,60,61,62]. Today, synthetic biology offers a wide range of approaches for the development of sensing, data communication, and target biochemical production platforms based on the cells of the microorganism. Using basic genome editing to cut some parts [63], synthetic genetic logic circuits [64] and the development of synthetic genomes [65] provides a huge variety in microbial cell redesign for the purposed goal. The engineered cells have already demonstrated possibilities in medical treatment, for example, in preventing the formation of a bacterial biofilm on the lungs [66]. Table 2 illustrates the synthetic biology approaches that can be considered for application in a microbial cell-based microrobot design.

The ability to produce chemicals inside the cell and secreting it in the necessary moments provide the opportunity to exclude external cargo in polymeric nano/micro capsules. It is necessary to mention that there are many drugs, especially anti-cancer, that are synthesized chemically, and there is currently no possibility to make them biosynthetically. Nevertheless, there are many prospective anti-cancer chemicals in the microorganisms, especially in microalgae [77]. *C. reinhardtii* microalga is very common as a biological base for the design of microrobots, as it can be seen from the previous section. This flagellated microalga is also very popular as an object of genetic engineering. Today, the application of CRISPR/cas9 methods for its genetic editing [78,79], the development of synthetic genetic circuits [80], and methods for effective transformation [81] have already been shown. The genome-wide screening of photosynthesis related genes in *C. reinhardtii* was provided in [82]. Additionally, as a photosynthetic organism, this microalga will produce oxygen on the light that is used to prevent hypoxia. All of these make *C. reinhardtii* one of the most promising candidates for a microrobot development using synthetic biology approaches.

The enhancement of cell-to-cell communications and the efficiency of taxis are related to the realization of computational procedures inside the cells [70,83,84]. Today, the most common approach is based on genetic logic circuits [13,64]. Based on the central dogma of molecular biology, this method operates with transcription factors, RNA-switches, and other components to realize logic gates with output in the form of specific protein synthesis. The number of logic gates in the single cell can be limited because of the usage of cellular recourse (amino acids, energy intermediates, etc.), but computation can be separated between different cells with chemical communications for signal transfer [83,84]. Thus, combinations of cells with different goals can be discussed for some applications [61]. For example, cell-sensors that focus on finding targets such as cancer in low attractant concentration conditions and then produce new attractants that could be enough to call cells—producers of the needed chemicals to fight against tumors.

The Keller–Segel framework for the mathematical modeling of chemotaxis does not discuss in depth the intracellular mechanisms of signal transmission and flagella rotation. The system biology of the intracellular mechanisms of chemotaxis and biofilm formation have been in the focus of a number of studies in recent years [19,24,85,86]. The engineering of chemotaxis receptors can force cells to react on a new chemical that is related to the target [75]. Thus, all intracellular mechanisms can stay the same without any modifications. The possibility to design or modify chemotaxis receptors is far from unlimited, but there are interesting perspectives in the search for new structures by using novel approaches based on the ALPHA fold system [87,88,89].

The special issue that should remain the center of attention of the scientific community is the safety of the discussed microrobots in all of their possible applications. Most issues and approaches related to this problem are the same as in the case of whole synthetic biology [90]. Here, we discuss in detail, one field called xenobiology. The term “xenobiology” means adding in the organism’s new building blocks based on artificial chemistry that is absent in the living cells [91]. This means the redesign of genetic code by adding new non-canonical nucleic acids called xeno-nucleic acids (XNA) as well as non-canonical amino acids (nnAAs). Thus, an expanding amount of biological information flow is taking place, which offers the possibility of designing new proteins [92,93]. Several approaches of stable XNA synthesis have already been presented [94,95]. XNA with eight nucleotides (four canonical and four xeno) has been synthesized and RNA-like molecules were processed from it [95]. In this approach, there is a significant opportunity to make a cell in which division is possible only in cases where xeno-nucleotides exist in the environment. This gives a strong instrument to control cell population [96]. It is important to note that such xeno-nucleotides should not be metabolized by the canonical biological objects. The same results can be obtained by using nnAAs in life-essential protein structures. The development of XNA-based insertion in the microorganisms as a basis for microrobot design could be the most reliable safety instrument that can strongly control its population during application.

Table 3 demonstrates the main tools related to synthetic biology and prospective ways of its application for the microbial cell based microrobot design. Generally, all tools can find an application. Deoxyribonucleic acid (DNA) synthesis can be used for the development of new or modified chemotaxis sensor genes, genes responsible for treatment chemical synthesis, etc. All methods of genetic engineering and editing can also be applied. It is necessary to mention mathematical modeling and simulation in all microrobot development stages including genetic engineering. Such computer aided design is common in other engineering fields and could significantly enhance the efficiency and safety of the microbial cell-based microrobots.

## 4. Current and Future Applications

The main field of the application of microrobots is in medicine [51]. The basic idea is to use cell motility to bring drugs as the cargo with high accuracy to the target—a place with pathology needs to be cured (e.g., its advanced drug delivery). In the case of microalgae or algae, an additional opportunity exists because of photosynthesis, which means that they can produce oxygen to fight against hypoxia [56]. Microrobots demonstrate a long-time operation and produce treatments as a reaction on the sensed molecule related to dysfunction [102]. A significant advantage of microrobots is the opportunity to bring different cargos in one target. Thus, requiring step-by-step components by adding multicomponent drugs can be used. This gives variability to the treatment depending on the health condition of the patient.

**Figure 3:**
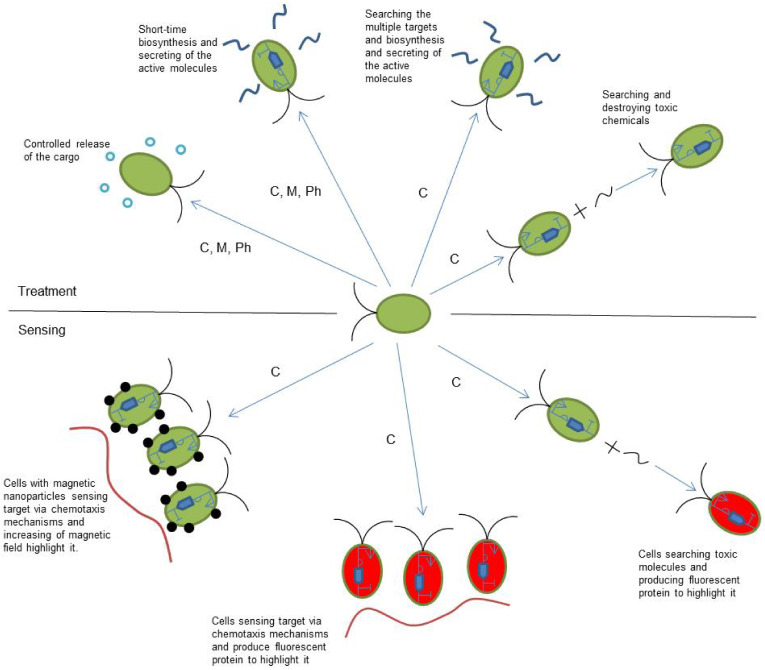
Possible applications of microbial cell-based microrobots for medical treatment and sensing. C, M, and Ph are the motility types that can be used for current applications and are chemotaxis, magnetotaxis, and phototaxis, respectively.

Search and identification of the target for further treatment uses the same basic idea as for cargo transportation. In this case, modified chemotaxis and the mechanism of the cells’ detection are necessary. Chemotaxis should be focused on those related to the target molecules. The magnetic nanoparticles can also be used as a detection factor. Increasing the number of cells will lead to an increase in the magnetic field parameters, which can be detected externally. Biosynthesis of the fluorescent molecules, triggered by the presence of attractive molecules [13,103], also can be taken into account as a possible detection factor [104]. Microorganism-based microrobots can realize search-and-destroy behavior to remove toxic molecules from the organism. This is a required modification of the chemotaxis receptors to react on the target toxic chemical as an attractant. The same idea can be used for sensing whether the cells will provide fluorescent protein biosynthesis in the case of contact with toxic molecules [105,106]. Figure 3 shows the possible applications of microbial cell-based microrobots for medical treatment and sensing.

The fields of application of microrobots are not limited by medicine. There are several options for environment monitoring and control as well as novel hybrid living materials (HLM):

1. Detection of toxic pollutants in the environment [107]. For example, the possibility of detecting arsenite by whole-cell biosensors has already been presented [108]. A combination of such sensors with motility and genetic logic circuits can be a promising approach for environmental monitoring.
2. The removal of toxic pollutants from the environment. As in the case of similar applications in medicine, microrobots can realize search-and-destroy behavior to remove toxic molecules. This requires modification of the chemotaxis receptors to react on the target toxic chemical as an attractant.
3. HLM with microorganisms that provide self-healing of the material and/or additional functionality such as sensing and the production of useful chemicals, air treatment, etc. [109,110,111]. In the case of the application of a microfluidic network that can provide efficient microorganism transport, microrobots can be a living part of an HLM.

Large-scale environmental systems can lead to an issue with the microrobot’s feedback control. For example, it can be difficult to control small numbers of the microrobots separated on the water body directly in the case of the application for toxic removal from a garden pond. In such situations, non-direct control of the chemical and biological parameters of the pond itself should be taken into account.

Microorganism-based microrobots are not limited by fluorescent protein synthesis as the output signal. There are possibilities to use luminescence or the biosynthesis of easy-to-detect chemicals [61]. The organics that can be detected via enzyme-based or microbial-based electrochemical sensing can be used. Such sensors are widely discussed nowadays for multiple applications [112,113].

The main advantages of microrobot applications for sensing are as follows:

- Crowd effect—the more cells via chemotaxis generate an output signal in some places that have a higher concentration of attractant. Thus, distribution and power of attractant sources theoretically can be displayed;
- Multiply parameter sensing—by using genetic logic circuits, it is possible to provide the biosynthesis of different fluorescent proteins as the output related to different combinations of the measured parameters in the media [13,14];
- Microrobot sensor networks—distribution of sensing duties between different groups of cells, each of them responsible for sensing its own group of parameters [61].

It is necessary to mention that in most cases, a single cell is a discrete or several-state sensor. For example, the cell can synthesize three fluorescence proteins depending on the combination of the input parameters [14]. Thus, it has four states: no fluorescence and three colors of fluorescence. To receive an analog signal, it is necessary to use multiple cells, as in the case of the crowd effect. Nevertheless, combination of controlled motility, especially chemotaxis, and the above-mentioned advantages show bright future for the use of microorganism-based microrobots in its application in the advanced sensing of a whole area.

## 5. Discussion and Conclusions

Each technology has limitations in terms of its application. In microorganism-based microrobots, these limitations are mostly related to the microorganism itself. Regarding toxic chemicals and other dangerous cells, it is necessary to mention that there are chemicals and cells that are dangerous for microorganisms. Although an immune reaction was not observed in the published papers, it does not mean that it cannot arise in other medical applications. The same issue concerns any environmental applications, where the number of chemicals and organisms that can potentially kill the microorganism’s cells in the microrobots are significantly higher. Another limitation is the dark side of the advantage—microscale systems are influenced by the number of factors related to the hydrodynamics and mass transfer. Such factors can be discussed as predictable in closed or partially closed environments, but not in open systems such as in the cases of application in environmental monitoring in water systems, for example, lakes and rivers.

The perspectives of microbial cell-based microrobotic technology is very wide and is strongly related to the extremely developing field of synthetic biology. Many of the usual techniques in this field of science approaches have not been applied in microrobotic design yet, which concern both functionality and safety. The XNA design and the addition of nnAAs in the cell of the microorganism can provide an effective switch that can turn off the microrobot in any out of control situation. Taking into account recent progress in the development of synthetic cells [65,114], and in the mathematical modeling of all intracellular processes [100,101], a design for the synthetic genome of the microrobots’ bacterial cells can be discussed in the near future.

In summary, one can say that microorganism-based microrobots can find wide application in medicine, environmental technology, and even in novel materials. It is very difficult to predict the innovation process, but there is a possibility that new applications will arise in the near future. It is clear that these types of microrobots will soon be common in practice.

## Figures and Tables

**Table 1:** Advantages and limitations of the microrobot’s motility types.

Motility Type	Speed without Cargo	External Stimulus	Advantages	Limitations	References
Chemotaxis	Around 20–50 μm/s	Attractant, but it can be an internal chemical synthesized inside the organism where the microrobot is applied	Can be realized without external stimulus. Well-known and can be discussed as a target for genetic engineering	Lower speed, more difficult behavior in comparison with other types of motility.	[15,38]
Phototaxis	Can be larger than 100 μm/s	Light needed as an external stimulus	Phototrophic microorganisms also produce oxygen that is helpful to prevent hypoxia. Can be to the light and out of light depending on the strain and light intensity.	Cannot work without light.	[16,41,49,50]
Magnetotaxis	Dependent on the strain and magnetic field parameters	A magnetic field is necessary	Can be compatible with MRI. Possible to induce by adding nanoparticles to the cells.	An external magnetic field is necessary. Artificial magnetotaxis is not inherited through the generations of the cells.	[52,54]

**Table 2:** Synthetic biology approaches that can be considered for application in a microbial cell-based microrobot design.

Synthetic Biology Approach	References	Possible Applications for Microrobots	Comment
Adding biosynthesis of new for the microorganism’s chemicals	[67,68,69]	Production of the necessary chemical that can be a drug against target illness	Replacing cargo with biosynthesis leads to the saving of this ability in throw-out generations.
Computations in the cells, genetic logic circuits, and based on those methods of cell-to-cell communications	[70,71,72]	Enhancement of quorum sensing and efficiency of chemotaxis	The more microrobots with some toxic anticancer cargo reach the target, the less negative impact they will have on the whole organism.
	[14,64,73,74]	Development of analysis of the received signal and generation of the answer based on the provided computation	Offers the possibility to make the behavior of the microrobot more complex and can add some additional chemical sensors to enhance efficiency in reaching the target.
Engineering of motility related mechanisms and sensors	[75,76]	Engineering receptors for the new attractant related to the targets, or modification of the chemotaxis pathway to fit it with new receptors.	Increasing the efficiency of chemotaxis, development of synthetic chemotaxis pathways related to the microrobot’s target.

**Table 3:** Synthetic biology tools and prospective ways of their application in microbial cell-based microrobotic engineering and design.

Synthetic Biology Tool	References	Possible Applications for Microrobotic Engineering
DNA assembly and DNA synthesis	[97]	Biosynthesis of chemicals for treatment, cell-to-cell communications, enhanced sensing, novel sensing molecules for chemotaxis
Genome editing	[78,79,97]	All applications where manipulation with genome required
Genetic circuits	[13,14]	Computation in cells, cell-to-cell communications, triggers, and switches
XNA assembly and integration	[94,95]	Safety, control of cell population
Biological parts/biobricks	[98,99]	Fast development of synthetic genetic circuits
Intracellular processes and cell behavior through mathematical modeling and simulations	[100,101]	Modeling intracellular processes and the behavior of developed microrobots, and the simulation of its application

## Data Availability

There are no additional data except those presented in the paper.

## Abbreviations

DNA	Deoxyribonucleic acid
DOX	Doxorubicin
HLM	Hybrid living materials
MRI	Magnetic resonance imaging
nnAAs	Non-canonical amino acids
RNA	Ribonucleic acid
XNA	Xeno-nucleic acid
